# Valorization of avocado seeds with antioxidant capacity using pressurized hot water extraction

**DOI:** 10.1038/s41598-022-17326-5

**Published:** 2022-07-29

**Authors:** Eng Shi Ong, Janelle Low, Joseph Choon Wee Tan, Su Yi Foo, Chen Huei Leo

**Affiliations:** Science, Math & Technology, Singapore University of Technology & Design, 8 Somapah Road, Singapore, 487372 Republic of Singapore

**Keywords:** Chemistry, Analytical chemistry, Green chemistry, Chemical biology, Metabolomics, Natural products

## Abstract

The pulp of avocado (*Persea Americana*) is widely consumed as the primary food source, while the seed is often discarded as food waste. Increased consumption of avocado would inevitably results in production of waste by-products such as avocado seeds, hence the ability to extract phytochemicals from such waste, and upcycling to potential nutraceutical products is of great interest. The overall aim of this study is to explore avocado seeds as potential functional food through the combined use of a green extraction method, chemical standardization and pattern recognition tools, and biological characterization assays. Specifically, this study utilized an organic solvent-free extraction method, pressurized hot water extraction (PHWE) to extract phytochemicals from avocado seeds and liquid chromatography mass spectrometry (LCMS) was used to identify the phytochemicals present in the avocado seeds. Our results demonstrated that avocado seed extracts have antioxidant activity and inhibited oxidative stress-induced metabolomics changes in endothelial cells, suggesting that avocado seed extracts have vasoprotective actions.

## Introduction

*Persea Americana* (avocado), are fruits commonly found in the various parts of the world, which are well-known to contain a variety of compounds^1^. Examples of compounds found in avocado include fatty alcohols, phenolic compounds with aromatic rings, sugar and sugar alcohols, furan and furanone derivatives and diterpenoids^2^. Hence, it is well-regarded that avocado are beneficial in supporting for cardiovascular health, weight management and healthy aging, leading to increase consumption of the fruit^1,3,4^. Due to increased consumption and processing of avocado, by-products and waste such as the skin and seeds are often discarded as waste. However, these waste products can be a rich source of bioactive compounds that can be developed as functional food ingredients; several studies have found the seeds to be rich in polyphenols with antioxidant properties^3,5–8^.

Extraction is one major step in getting bioactive compounds and other ingredients from plant materials. Based on various reports, the extraction of bioactive constituents from avocado seeds required the usage of organic solvents such as methanol^3,5–8^. The contributing effects of various extraction methods on the physiochemical characteristics, antioxidant activities, and others of avocado seeds were important factors. To reduce or eliminate the usage of organic solvents, modern extraction techniques including supercritical fluid extraction, subcritical water extraction or pressurized hot water extraction (PHWE) and others, have been widely reported for the extraction of target compounds from a wide variety of plant and food sources^9,10^. Chemical standardization with the determination of selected compounds, chemical fingerprint with liquid chromatography mass spectrometry (LCMS) with pattern recognition tools, such as principal component analysis (PCA) are commonly used to evaluate the quality of the plant-based extracts. Additionally, a combination of chemical standardization, antioxidant and biological assays would be critical to ascertain the quality of the end product^11^.

The aim of this current work is to evaluate antioxidant properties of chemical compounds from avocado seeds. The biological activities of the avocado seeds would be characterized using antioxidant assays and lipidomics in endothelial cells. Lipidomics, also known as redox lipidomics, presented itself as a communication language that provided useful information in the study of biological functions, through the analysis of lipids, peroxidized phospholipids and other small molecules, which served as important markers for cellular stress and apoptosis^12–14^.

## Results and discussion

**Figure 1:**
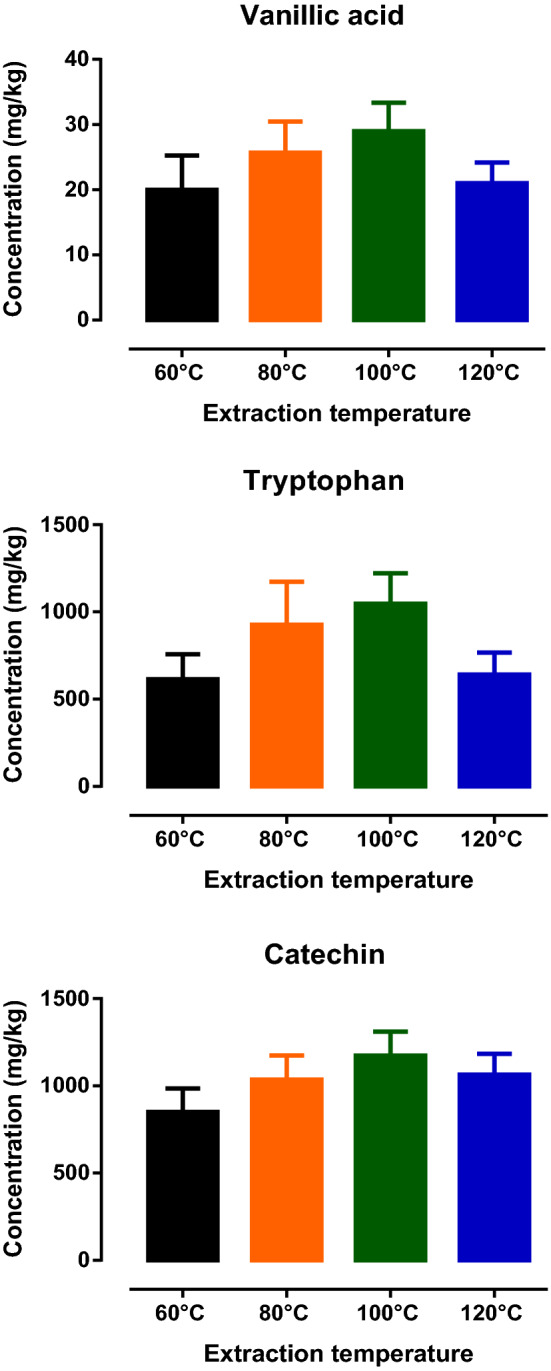
Amount of vanillic acid, tryptophan and catechin (mg/kg) detected in avocado seed extracts from PHWE at 60 °C, 80 °C, 100 °C and 120 °C with LC/UV by external standard calibration (n = 3).

It was well-noted that one of the most critical parameters that affected the performance of PHWE was the extraction temperature^9,10^. Hence, the initial experiment aimed to extract avocado seeds at four different temperatures, 60, 80, 100 and 120 °C using PHWE and compared to sonication with methanol. Marker compounds such as catechin, tryptophan and vanillic acid present in the seed extracts were monitored using both LC/UV and LC/MS. Although an increasing trend for the amount of compounds extracted from the seeds was observed from 60 to 100 °C, no significant differences between the amount of compounds from the extracts were observed between the four different extraction temperatures (Fig. 1). The extraction efficiency of PHWE was compared to sonication with methanol. It was noted that the amount of target compounds obtained from the PHWE at 80 °C was found to be comparable or higher than sonication with methanol (Table 1). At the same time, higher variability was observed for the extraction of target compounds present in avocado seeds for sonication with methanol as compared to PHWE. Using sonication with methanol, it was proposed that the target compounds may not be able to partition themselves from the sample core into the extraction fluid, resulting in lower extraction efficiency compared with PHWE.

**Table 1:** Comparison of amount of vanillic acid, tryptophan and catechin present in two different batches of avocado seed extracted using PHWE at 80 °C (n = 6) and methanol (n = 3).

Standards	Concentration (mg/kg)			
	First Batch		Second Batch	
	PHWE at 80 °C	MeOH Extracts	PHWE at 80 °C	MeOH Extracts
Vanillic Acid at 260 nm	20 ± 5	ND	150 ± 6	55 ± 55
Trytophan at 280 nm	960 ± 300	130 ± 100	900 ± 200	50 ± 50
Catechin at 277 nm	1200 ± 200	750 ± 700	1280 ± 250	220 ± 550

**Figure 2:**
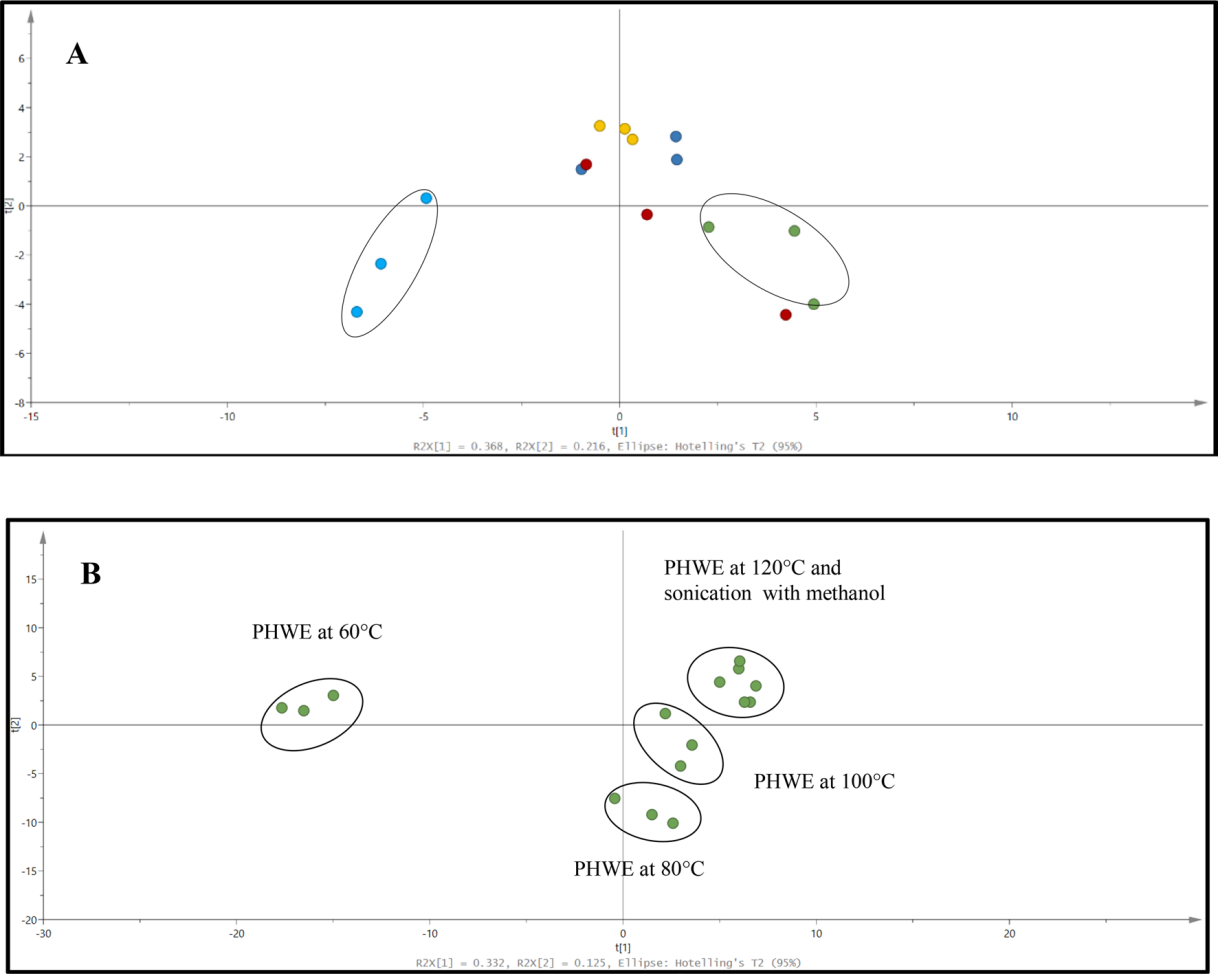
PCA score plot of (**A**) LC/UV profile and (**B**) LC/MS profile of avocado seed extracts. (**A**) PCA score plot of LC/UV profile detected at 254 nm, Green: PHWE at 60 °C, Dark blue: PHWE at 80 °C, Red: PHWE at 100 °C, Yellow: PHWE at 120 °C & Light blue: sonication with methanol. (**B**) PCA score plot of LC/MS profile of avocado seed extracts by PHWE at 60 °C, 80 °C, 100 °C, 120 °C and sonication with methanol.

Based on the chromatographic profiles from LC/UV and targeted ions from LC/MS obtained at different extraction temperature, the normalized data for selected peaks of each sample were analysed using pattern recognition tools such as PCA^11,15^. Based on the PCA score plot, distinctive chemical profiles were observed from the seeds extracted at 60, 80, 100 and 120 °C (Fig. 2). While the two may seem synonymous, seeds extracted at 60 and 120 °C were observed to have a completely distinct fingerprint from the extracts at 80 and 100 °C. In conjunction, the PCA score plot from the chromatograms obtained from LC/UV (Fig. 2A) illustrated a complementary formation which portrays a degree of similarity between the characteristic profile of seeds extracted at 80 and 100 °C, while extracts of sonication with methanol and PHWE at 120 °C were quite distinct. The distinctive clusters from various extraction conditions were also observed with the independent peaks obtained from LC/MS (Fig. 2B). From this set of data, the variation in the profile of these seeds were most likely due to the different extraction parameters that resulted in the production of a varying amount of phytochemical content. The holistic nature of botanical extracts creates a challenge in establishing the quality for the product and monitoring of a single compound from extraction. Furthermore, although we have not done any direct measurements of unwanted compounds extracted from the avocado seeds, we did not observe any cell death or signs of cellular stress based on the concentration of extracts tested and duration of treatment. Hence, it is logical to hypothesized that the presence of these unwanted compounds (if any) is negligible to cause any negative effects in this study. Hence, chemical fingerprinting provided an approach for the quality assessment of the extracts obtained^11,16^.

**Figure 3:**
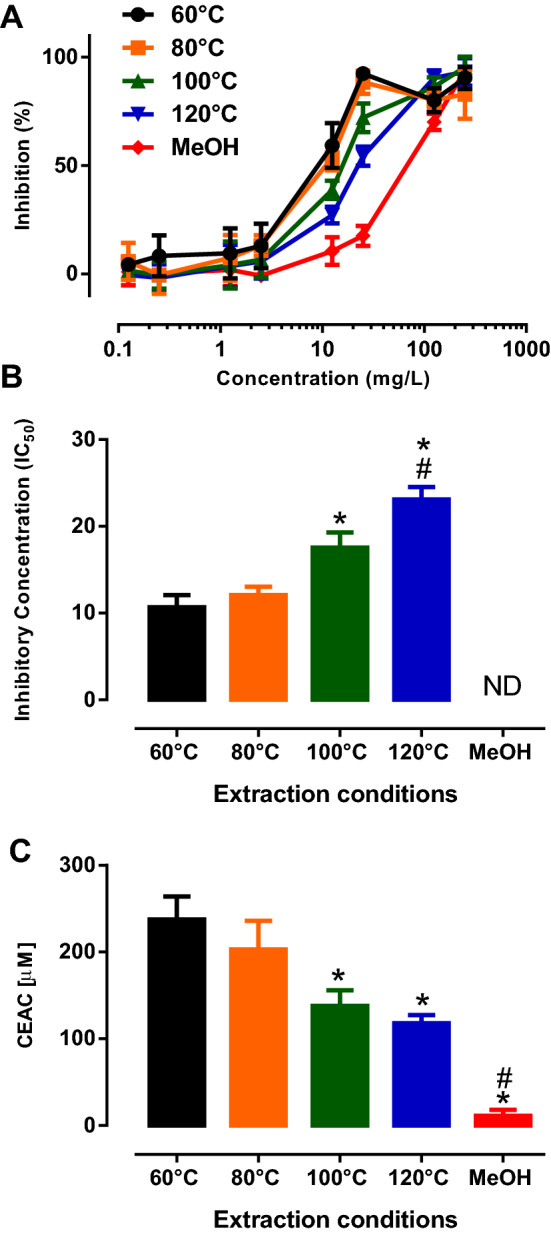
Antioxidant activity of avocado seed extracts for PHWE and Sonication with methanol. (**A**) Concentration response curve of avocado seed extracts against DPPH inhibition, (**B**), Inhibitory concentration (IC_50_) of avocado seed extracts derived from Fig. 3A, (**C**) Vitamin C Equivalent Antioxidant Capacity (CAEC) of avocado seed extracts. *significantly different from 60 or 80 °C; #significant different from 100 or 120 °C, *P* < 0.05, (1-way ANOVA, Tukeys’s post-hoc test). Data is presented as mean ± SD, n = 3–4.

Numerous earlier reports have demonstrated that avocado seeds extracts obtained through organic solvent (ethanol or methanol) extraction have strong antioxidant capacity^5–8,17^. From our earlier works^9–11^, it was also noted that the monitoring of bioactive and targets compounds provided means to evaluate the extraction efficiencies of the proposed method of extractions. Therefore, in this study, we evaluated the antioxidant capacity of avocado seed extracts from various PHWE temperatures and methanol extraction. Consistent with previous studies^5–8,17^, avocado seed extracts caused a concentration-dependent inhibition of DPPH, indicating that avocado seed extracts have strong antioxidant capacity (Fig. 3A). Furthermore, a gradual rightward shift of the concentration response curves of the avocado seed extracts as the extraction temperature increased from 60 to 120 °C. Interestingly, responses to methanol extraction had the most rightward shift of the concentration response curves, suggesting that it has the least potent antioxidant activity in comparison to avocado seeds extracted using PHWE (Fig. 3A). When the antioxidant activity was evaluated using different temperature of PHWE, it was evident that the extracts at 60 and 80 °C have significantly (*P* < 0.05) lower IC_50_ values for DPPH inhibition when compared to PHWE at 100 and 120 °C. Furthermore, it was also noted that there was no statistical difference in DPPH inhibition between at 60 and 80 °C (Fig. 3B). Consistent with the DPPH assay, 60 °C and 80 °C seed extracts also revealed significantly higher CEAC values when compared to either higher temperature of PHWE (100 and 120 °C) or methanol extraction (Fig. 3C). Taken together, our DPPH and ABTS assay data suggested that 60 and 80 °C seed extracts have the most potent antioxidant capacity. Although distinctive chemical fingerprint was observed for PHWE at the various extraction temperature, and sonication with methanol (Fig. 2), it appears that certain target compounds seem to be present in higher concentration at a higher applied temperature (Fig. 1). However, this may contradict with our findings with the DPPH and ABTS assays, where the antioxidant capacity was stronger at the lower extraction temperatures. Hence, it was proposed that the higher applied temperature might have degraded some of the bioactive compounds present in the seed samples, or there could be the presence of higher levels of non-targeted or unknown phytochemicals with strong antioxidant activity at the lower extraction temperatures. As seen in our earlier work^11^, a combination of chromatographic analysis, pattern recognition tools, antioxidant assays provided a useful approach for the monitoring of extraction efficiencies at different applied temperatures. While this approach may not necessarily account for the synergistic effects that may be present in botanical extracts with their associated antioxidant capacity^11^, nonetheless, in this study, the combination of these chemical and bioassay allowed the optimisation of PHWE of avocado seed extract at 80 °C, which would be subjected to further experimentation.

**Figure 4:**
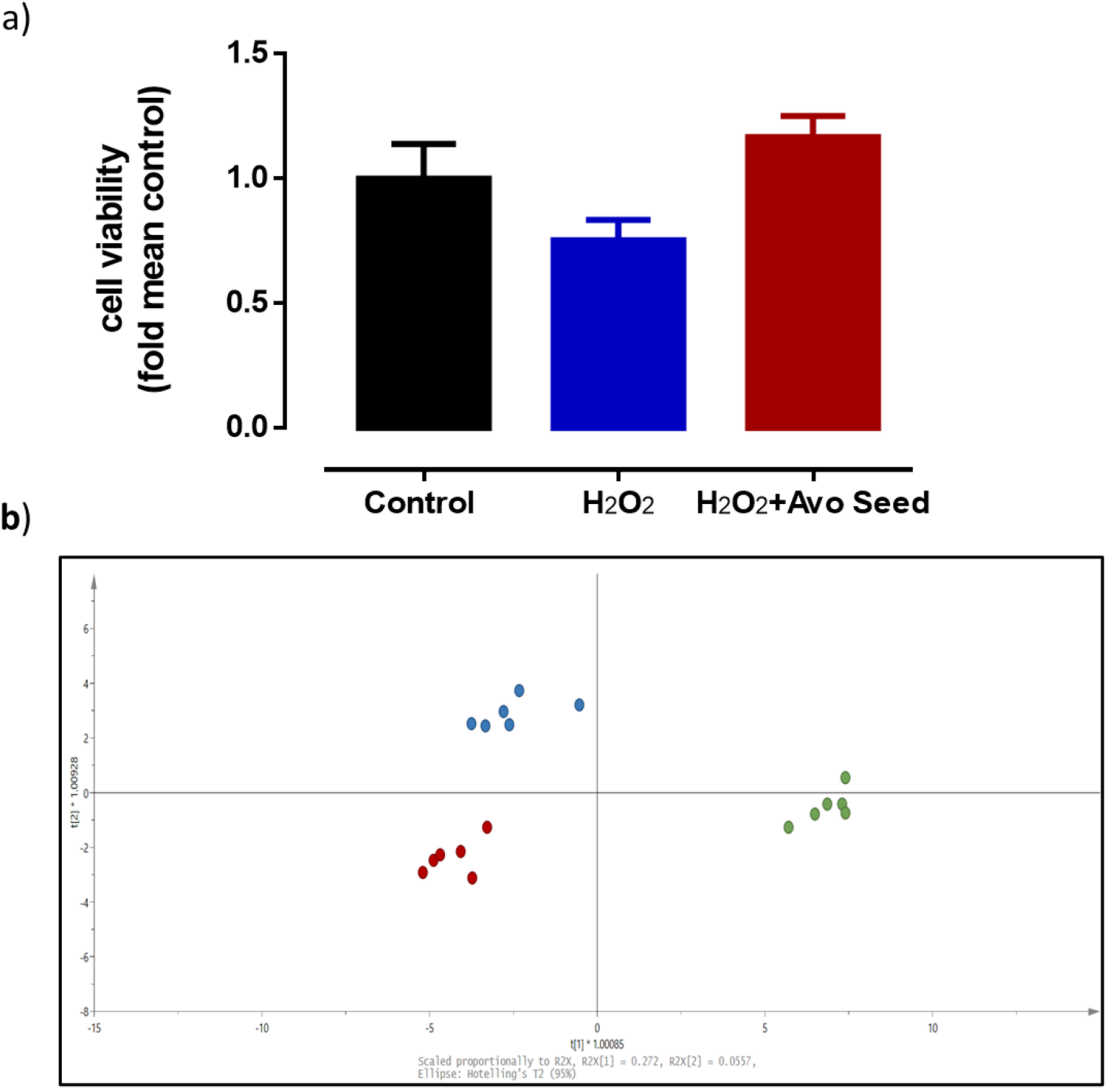
Effects of avocado seed extracts treatment in HMEC-1 cells under oxidative stress conditions. (**a**) Cytoprotective effects of avocado seeds extracts on H_2_O_2_-stimulated HMEC-1 cells. (**b**) OPLS score plot of LC/MS lipids profile of avocado seeds extracts treated cells, Green: Control, Blue: H_2_O_2_ only, Red: H_2_O_2_ and avocado seeds extracts. Data is presented as mean ± SD, n = 4–6.

Although chemical based assays (DPPH and ABTS) are convenient methods to determine the antioxidant activity of any botantical extract, it remain inconclusive if the antioxidant activity has any biological relevance in a cellular system. Hence, the antioxidant effect of the avocado seed extracts was investigated using oxidative stress-induced cytotoxicity. Endothelial cells that were exposed to H_2_O_2_ had lower cell viability in comparison to untreated control cells. However, the co-incubation of avocado seed extract with H_2_O_2_ appeared to have increased cell viability, although it failed to reach statistical significance (*P* = 0.068) (Fig. 4a). This finding was suggestive that the avocado seed extract was able to reduce H_2_O_2_-induced cytotoxicity, in part through its antioxidant activity.

Lipids and other metabolites are small molecules that have many key biological functions, including structural components of cell membrane, energy storage and others^13,14,18^. Oxidative modifications of lipids and other biomolecules induced by reactive oxygen species have been implicated in the progress of many diseases^19–21^. Specifically, oxidative stress-induced endothelial dysfunction is well-established as an initial factor for cardiovascular diseases^22–24^. The presence of oxidative stress causes changes in the lipid profile and antioxidants from natural sources may exert their protective roles either directly or indirectly in the physiological defence network to inhibit oxidative modification of lipids^19,25–27^. Therefore, the potential protective effects of avocado seed extracts were further characterized via metabolite profiling of several lipid markers in endothelial cells. Based on the OPLS score plot, distinctive clusters and a marked alteration of metabolites in the endothelial cells between various groups was observed (Fig. 4b). The current data was consistent with our earlier work where distinctive clusters were observed after the cells were treated with H_2_O_2_^28^. Based on the VIP values in Fig. S1 (supporting information), a number of metabolites were found to be different between the control group versus the H_2_O_2_ treatment, control versus H_2_O_2_ treatment/seed extract treatment group, and H_2_O_2_ treatment versus H_2_O_2_ treatment/seed extract treatment group. Although it was reported that OPLS may aggressively force the separations between 2 experimental groups^29^, we have observed that OPLS will not be able to force the separation between two groups that may not have the distinctive features^30^.

From Fig. 4b and Table 2, it was noted that a shift in the metabolite profile was observed after the treatment of endothelial cells with H_2_O_2_. However, using the current method, a number of metabolites that were found to be differentially expressed cannot be positively identified. Lysophosphatidylcholine or lysolecithins such as LPC18:2 and others are known to upregulate genes involved in cholesterol synthesis and involvement in fatty acid oxidation^31^. It may modulate inflammatory chemokine expression from endothelial cells, increase oxidative stress and inhibit endothelial cell migration, proliferation and dysfunction^4,14,31–34^. Indeed, in this study, the level of LPC C18:2 was significantly increased after the cells were treated with H_2_O_2_, suggesting possible endothelial cell damage. However, the level of LPC18:2 remained the same after the addition of the avocado seed extracts to H_2_O_2_ treatment. Related to lipid metabolism, metabolites such as choline and betaine are the basic constituent of lecithin in animal organs and methyl donor involved in various metabolic processes. Under oxidative stress condition, it was proposed that H_2_O_2_ would lead to a decrease in basic constituents such as choline and betaine, causing a higher production of LPC18:2 in endothelial cells^31^. Although statistically insignificant, the levels of choline, betaine glycerphosphocholine and acetylcarntine appeared to be lower after treatment with H_2_O_2_ in comparison to untreated control. In endothelial cells co-incubated with H_2_O_2_ and avocado seed extracts, the amount of choline, betaine, glycerphosphocholine and acetylcarnitine were found to be in the trend of restoring to control levels. Hence, the avocado seed extracts treatment seemed to be able to partially restore the levels of choline, betaine glycerphosphocholine and acetylcarntine in H_2_O_2_-induced oxidative stress damage to endothelial cells.

**Table 2:** Normalised peak areas of lipids and others present in HMEC-1 cells from control, H_2_O_2_, and H_2_O_2_ and avocado seed extracts, Data is presented as mean ± SD, n = 6. RT: retention time.

m/z/RT	Compound identified	Control	H_2_O_2_	H_2_O_2_ & Avocado seed extract
104/0.5	Choline	0.095 ± 0.033	0.070 ± 0.023	0.0855 ± 0.0281
118/0.6	Betaine	0.006 ± 0.002	0.00476 ± 0.00045	0.00450 ± 0.00110
258/0.6	Glycerphosphocholine^3^**	0.0111 ± 0.0052	0.00596 ± 0.00068	0.00766 ± 0.000679
524/10.6	LPC C18:0	0.00621 ± 0.00295	0.0103 ± 0.0018	0.0113 ± 0.0018
204/0.6	Acetylcarnitine	0.00621 ± 0.00295	0.00387 ± 0.00046	0.00547 ± 0.00158
520/8	LPC C18:2^1^**^,2^**	0 ± 0	0.00279 ± 0.00022	0.00280 ± 0.00043
448/6.7	GDCA	0.000114 ± 0.000256	0.000288 ± 0.000048	0.000308 ± 0.000051
371/5.5	Thromboxane B1^1^**	0 ± 0	0.000141 ± 0.000114	0.000089 ± 0.000145
255/11.3	Palmitic Acid	0.0005 ± 0.0003	0.000586 ± 0.000111	0.000655 ± 0.000211
391/8	DA^2^**^,3^**	0 ± 0	0.000094 ± 0.000146	0.000371 ± 0.000127
498/5.8	TDCA^2^**^,3^**	0.00014 ± 0.00013	0.0001400 ± 0.000030	0.000219 ± 0.000024

^1^Denotes significant different between the control group and the H_2_O_2_ group.

^2^Denotes significant different between the control group and H_2_O_2_ and avocado seed extract treatment group.

^3^Denotes significant different between the H_2_O_2_ group and H_2_O_2_ and avocado seed extract treatment group.

*Denotes a *p* value of less than 0.05, ** denotes a *p* value of less than 0.01, 1-way ANOVA, Tukey’s post-hoc test.

**Figure 5:**
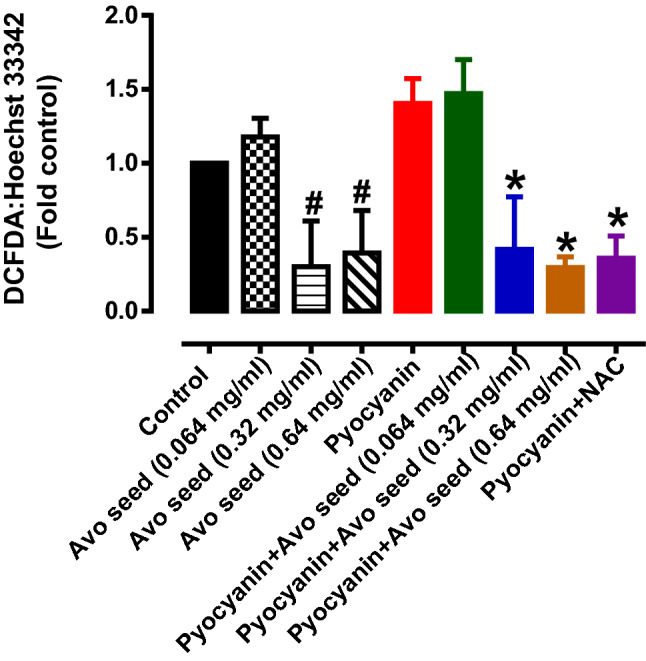
Intracellular reactive oxygen species measurement of avocado seeds extracts treatment of HMEC-1 cells under basal and pyocyanin-induced oxidative stress. # Significantly different to control; * Significantly different to pyocyanin, *P* < 0.05, (1-way ANOVA, Tukeys’s post-hoc test). Data is presented as mean ± SD, n = 3–6.

Bile acids such as deoxycholic acid (DA), taurodesoxylcholic acid (TDCA) and others, are a family of molecules that are involved in key systemic functions in human cells. The bile acids stated above reportedly play a key role as signalling molecules by modulating cell growth, gene expression and lipid metabolism^35–37^. Specifically, it was also demonstrated that bile acids possess vasoprotective action through their modulation of endothelium-derived nitric oxide production and activity^38^. In this study, we demonstrated that bile acids such as DA and TDCA were significantly upregulated in cells treated with H_2_O_2_ in comparison to untreated control, suggesting that this could be a compensatory mechanism to protect the endothelial function in response to the oxidative stress damage. Surprisingly, in endothelial cells that were co-incubated with H_2_O_2_ and avocado seed extracts, there was a further significant increase in the production of bile acids when compared to H_2_O_2_ alone (Table 2). Natural product in plants have been described to interact with bile acids and may affect bile acid metabolism^36^. Perhaps avocado seed extracts, through increasing bile acid production, may offer enhanced vasoprotection against oxidative stress damage in endothelial cells. Thromboxane B1, is a lipid mediator that is derived from the cyclooxygenase pathways, which is an important mediator involved in vasoconstriction and contributor to endothelial dysfunction^22,39–41^. Indeed, in this study, the levels of thromboxane B1 were significantly increased after the cells were treated with H_2_O_2_. After the addition of the avocado seed extracts with H_2_O_2_, the level of thromboxane B1 was decreased when compared to H_2_O_2_ alone, although it failed to reach statistical significance. Consistent with the metabolomics data, direct measurement of reactive oxygen species in HMEC-1 was also determined under baseline or oxidative stress-induced (pyocyanin) conditions (Fig. 5). Under both conditions, avocado seed extract treatment significantly reduced the levels of intracellular reactive oxygen levels in HMEC-1 cells when compared to control, indicating that the antioxidant capacity of avocado seed was biologically activity (Fig. 5). Taken together, the antioxidant and cytoprotective effects of the avocado seed extracts against oxidative damage appears to be involved in reducing thromboxane B1 levels and increasing the production of bile acids, which are known to possess vasoprotective function through the nitric oxide pathway^38^.

In conclusion, this study demonstrated that avocado seeds extracted using PHWE yielded different chemical profiles when compared to other method of extraction. Despite some differences in the chemical profile, the bioactive compounds present in the avocado seed extracts from PHWE were biologically active. Specifically, avocado seed extracts were reported to have potent antioxidant activity, which inhibited oxidative stress-induced alteration to several key metabolites in endothelial cells. Besides the biological function of the avocado seed extracts, PHWE method was also reported to be more cost effective in manufacturing and environmentally sustainable, as water has lesser environmental impact in comparison to ethanol or other organic solvent based extraction^42,43^. Therefore, PHWE is an environmentally friendly and cost-effective technique for the valorization of biologically active natural products from avocado seed, which may be beneficial for maintaining vascular health, particularly under oxidative stress conditions.

## Materials and methods

### Chemicals and materials

All commercial food imports that enter Singapore must come from accredited food establishments in approved countries and only traders who are licensed or registered with Singapore Food agency (SFA) can bring in commercial shipments of food (https://www.sfa.gov.sg/food-import-export/commercial-food-imports). In this study, fruit vendors kindly donated Hass avocado seeds waste for research purposes, hence the avocado seeds used are not species that are at risk of extinction. Based on the avocado seeds collected, the experimental research conducted in this study comply with relevant institutional, national, and international guidelines and legislation for research on plant material. The seeds were then dried at 40 °C in a laboratory oven for 24 h. After drying, the samples were crushed using mortar and pestle, and sieved individually to separate into following particle sizes: below 0.3 mm, between 0.3 and 2.8 mm, and above 2.8 mm. Chemical analysis was performed on fine samples with particle size below 0.3 mm. All chemicals and solvents used throughout the experiments were of standard analytical grade and purchased from Sigma-Aldrich (Singapore), unless otherwise stated.

### PHWE system and sonication with methanol

For the extraction of active compounds from each sample, a laboratory system was assembled similar to our earlier report^9^. A stainless-steel extraction cell (250 × 10 mm i.d.), an isocratic Shimadzu LC10 series pump (Kyoto, Japan), and a DVS 402 constant temperature oven (Hewlett Packard 5890 Series II) were used to assemble the proposed system. A stainless-steel tubing 1/16 in. o.d. and 0.18 mm i.d. was used for all connections. Ground samples of 0.5 g sample was weighed accurately into 50 ml tubes. A small proportion of sand was mixed with the sample before loading into the extraction cell. Flow rate of the pump was set at 1.2 ml/min and time for extraction was 40–45 min. The pressure recorded was 10–15 bar as indicated by the pump. For the optimization experiments, different temperatures at 60, 80, 100 and 120 °C were used. For chemical assay, the extract was used without any further treatment. For others, the extracts obtained from 0.5 g of original samples to 50 ml of water were dried off. The residue was dissolved in water and diluted for subsequent 2,2-diphenyl-1-picrylhydrazyl (DPPH), 2,2’-azinobis-(3-ethylbenzothiazoline-6-sulfonate) (ABTS) and cytoprotective assays. For sonication, 0.5 g of sample and 3 × 15 ml of methanol were added. The extraction was allowed to continue for 3 × 15 min. The extract obtained from 0.5 g of original sample to 15 ml of methanol were dried. The dried extract was dissolved in water and diluted for subsequent DPPH and cytoprotective assays.

### LC/UV/MS profiling of avocado seed extracts samples

LC/UV/MS was used to determine the phytochemical characteristic profile in different avocado seed extracts as previously described^11,28^. A C18 reverse phase High Performance Liquid Chromatography (HPLC) column (Zorbax SB-C18-3.5 microns, 4.6 × 100 mm) was used for compound separation. Flow rate was set at 0.25 mL/min while the column oven temperature was 40** °C**. The injection volume was 5 µL and concurrently separated into both HPLC (Shimadzu Nexera X-2) and LC–MS (Shimadzu LCMS-8050) machine. A gradient elution consisting of (A) 0.1% of formic acid in water and (B) 0.1% of formic acid in acetonitrile with the gradient parameter was performed in a negative mode ([M^-^H^-^]) at: 0.10 min 10% of B, 10 min 100% of B, 10.10 min 10% of B, and 15.10 min 10% of B, which was used to re-equilibrate the column to initial conditions. The MS Electrospray Ionisation (ESI) was operated with a nebulizing gas flow of 2.8 L/min, interface temperature of 300** °C** with a heating and dry gas flow of 9 L/min. On the other hand, the UV detector was set at 254 and 280 nm. The water-based extracts were filtered through a 0.2 µm filter into vials and placed into the autosampling rack for compound analysis. For the determination of catechin, tryptophan and vanillic acid, external standard calibration was used. The injection repeatability of the standards prepared was obtained found to be less than 2% (RSD, n = 6). Linearity was established from 0 to 100 g/L (R^2^ > 0.99).

For normalization to constant sum, the measured peak intensity for the reading of each sample derived from various sources of seed extracts was normalized prior to retrieving the average of the 3 sample readings. For LC/MS, the peak intensities that corresponded to the various molecular weight (m/z) were normalized within each sample to the total peak intensity of the sample. Normalization was performed to address the differences in concentration. Additionally, the normalized data for each sample was analyzed using the PCA scores plot for the selected peaks. For PCA, the data was condensed to 2 principal components to describe maximum variation in data points. From the PCA scores plot, observations can be made on clusters, trends and outliers.

### Antioxidant activity assays

The antioxidant activity of the avocado seed extracts was determined using DPPH and ABTS assays as previously described^11^. To determine the effect of extraction temperature on antioxidant capacity, samples were extracted at different temperatures (60, 80, 100 and 120 °C). The blank, control and various concentrations of the avocado seed extracts (0.4 µg/ml to 0.4 mg/ml) from different temperatures were prepared into the 96-well plate. Subsequently, excluding the blank, DPPH solution (100 µM) was added into each well for 30 min incubation at 37 °C. All experiments were performed in triplicates. Ascorbic acid (10 mM) was used as a positive control. The absorbance at 517 nm was measured using a spectrophotometric plate reader (Thermofisher Multiskan GO). For the ABTS assay, a free radical solution (5 mL) consisting of ABTS• + was prepared by adding 88 µL of potassium persulfate stock solution (140 mM) to ABTS solution (7 mM) and stored in the dark for 16 h. The solution was diluted 50-fold in distilled water to give an absorbance of approximately 0.80–0.70 at 734 nm. Several dilutions (100 μM to 1 mM) of vitamin C were prepared for the generation of the standard curve, which would produce between 5 and 50% inhibition of the blank absorbance (ABTS• + alone). The different avocado seed extracts (0.5 mg/ml) were also prepared. Three replicates of each sample were measured by adding 20 μL of each sample to the 96-well microplate containing 180 μL of ABTS• + solution. The plate was incubated at room temperature for 30 min. The Vitamin C equivalent antioxidant capacity (CEAC) value of the samples was calculated using the equation obtained from the linear regression of the standard curve substituted of absorbance at 734 nm values for the sample.

### Cell-culture

Dermal microvascular endothelium (HMEC-1) cells derived from newborn males were purchased from American Type Culture Collection (Manassas, Virginia, USA). HMEC-1 was cultured in MCDB-131, consisting of 20% FBS, 10–20 ng/mL of Recombinant Human Epidermal Endothelial Growth Factor (EGF), 10 µM of L-glutamic acid (Thermofisher Scientific, Singapore) and maintained at the incubation temperature of 37 °C at 5% CO_2_. The cells were seeded into the 6-well plate and left to adhere and proliferate for 24 h. Once the cells reached 80–90% confluency, cells were serum-starved (2% FBS) and used for subsequent experiments^28^.

### Cytotoxicity and metabolite profiling of cell extracts

Cellular cytotoxicity was induced by exposing the cells with reactive oxygen species (0.1 mM of H_2_O_2_). To determine if avocado seed extracts were able to alleviate cell cytotoxicity, the cells were co-incubated with avocado seed extracts (14 µg/ml) for the treatment duration in 37 °C, 5% CO_2_ incubator. For H_2_O_2_ incubation, the cells were treated for 4 h in duplicates. After 4 h of incubation, the cells were trypsinized and determined for cell viability using an automated cell counter (Biorad, Singapore). The % cell viability was calculated using the formula [(C_a_/C_c_) × 100%], where C_a_ stands for population of cells present in treated cells and C_c_ refers for the population of cells present in the control set-up.

Lipid extraction and analysis was performed in accordance to a previously reported article with minor modifications^12,28^. The treated cells were removed from the freezer and left to thaw at r.t.p (room temperature and pressure) for 5 min. A mixture of 1 mL of chloroform: methanol (3:1) was added to each 1.5 mL microcentrifuge tube containing the cells. The samples were vortex briefly. Then, the samples were centrifuged for 5 min at 10,000 rpm. The supernatant was transferred into a new microcentrifuge tube and placed in a vapor centrifuge for 30 min at 45 °C. After which, 200 uL of methanol was added to the dried sample and placed in a 3 ml scintillation vial with a 250 µL inserts prior to analysis on LC/MS. The LC/MS analysis was used to identify standard metabolites to confirm the cytoprotective results obtained. For the analysis of lipids and metabolites, a C18 reverse phase HPLC column (Zorbax SB-C18- 3.5 microns, 4.6 × 100 mm) was used. The flow rate was 0.25 mL/min while the column temperature was set at 40 °C. The injection volume was loaded into the system at 5 µL and concurrently separated into both LC–MS (Shimadzu LCMS-8050). The gradient elution involved a mobile phase consisting of (A) 0.1% of formic acid in water and (B) 0.1% of formic acid in acetonitrile with the gradient parameter performed at: 0.10 min 10% of B, 10 min 100% of B, 10 min 10% of B, and 15 min 10% of B, which was used to re-equilibrate the column to initial conditions. The MS Electrospray Ionisation (ESI) was operated with a nebulizing gas flow of 2.8 L/min, interface temperature of 300 °C with a heating and dry gas flow of 9 L/min.

For normalization to constant sum was performed as described earlier. The fatty acids, lipids and other small molecules were identified based on our earlier work and comparison with reference standards^12^.

### Detection of intracellular reactive oxygen species

Levels of intracellular reactive oxygen species were measured using the cell-permeant 2',7'-dichlorodihydrofluorescein diacetate (H_2_DCFDA) dye in accordance to manufacturers’ protocol (Caymen Chemicals, #601,520) with the following modifications. Briefly, HMEC-1 were exposed to either Hank’s balanced salt solution (blank) or 10 µM of DCFDA for 30 min. In some wells, the cells were treated with avocado seed extracts or N-Acetyl-L-cysteine, which served as a positive control. After 30 min incubation, 10 µL of pyocyanin was added to wells containing in either the absence or presence of avocado seed extracts to induce reactive oxygen species production in the cells. After 1 h of incubation, the cells were washed with Hank’s balanced salt solution to remove excess dye. Subsequently, the cells were counter-stained with Hoechst 33,342 (5 µg/ml) for 15 min. Following this, the cells were wash and the fluorescent signal for each dye were measured using the Varioskan LUX plate reader (Thermoscientific, Singapore).

### Statistical analysis

Soft Independent Modelling by Class Analogy (SIMCA) software was used to generate the PCA plots. Group mean values were compared using one-way ANOVA to test for statistically significant differences between different extracted temperatures of avocado seed extracts with post-hoc analysis using Tukey’s test. Concentration-response curves for DPPH inhibition were computer fitted to a sigmoidal curve using nonlinear regression (Prism version 5.0, GraphPad Software, San Diego, CA, USA) to calculate the sensitivity of DPPH inhibition for extract (IC_50_)^41,44^. All data were presented as mean ± SD. *P* < 0.05 was considered statistically significant.

### Ethics approval

Ethical review and approval were waived for this study. This study uses non-identifiable and commericially available cell line and does not involve the use of any humans or animals subjects.

## Supplementary Information

Supplementary Information.

## Data Availability

The data presented in this study is available in the article and Supplementary Materials.
