# Supplementary material for: Valorization of avocado seeds with antioxidant capacity using pressurized hot water extraction
Source: Sci Rep. 2022 Jul 29;12:13036. doi: 10.1038/s41598-022-17326-5 (PMC9338084; doi:10.1038/s41598-022-17326-5)
Supplement: Supplementary file 1 — Supplementary Information. [file 41598_2022_17326_MOESM1_ESM.docx]

**Supplementary Materials**

**Valorization of Avocado Seeds with Antioxidant Capacity using Pressurized Hot Water Extraction**

Eng Shi ONG^1,*^, Janelle LOW^1^, Joseph Choon Wee TAN^1^, Su Yi FOO^1^, Chen Huei LEO^1,^

^1^Science, Math & Technology, Singapore University of Technology & Design, Singapore 487372

**Corresponding Author:**

Dr Eng Shi ONG,

Singapore University of Technology and Design,

8 Somapah Road, Singapore 487372, Republic of Singapore

E-mail address: engshi_ong@sutd.edu.sg

Tel.: +65 6499 4513. Fax: +65 67795161.

**Supplementary Figure 1**

A)


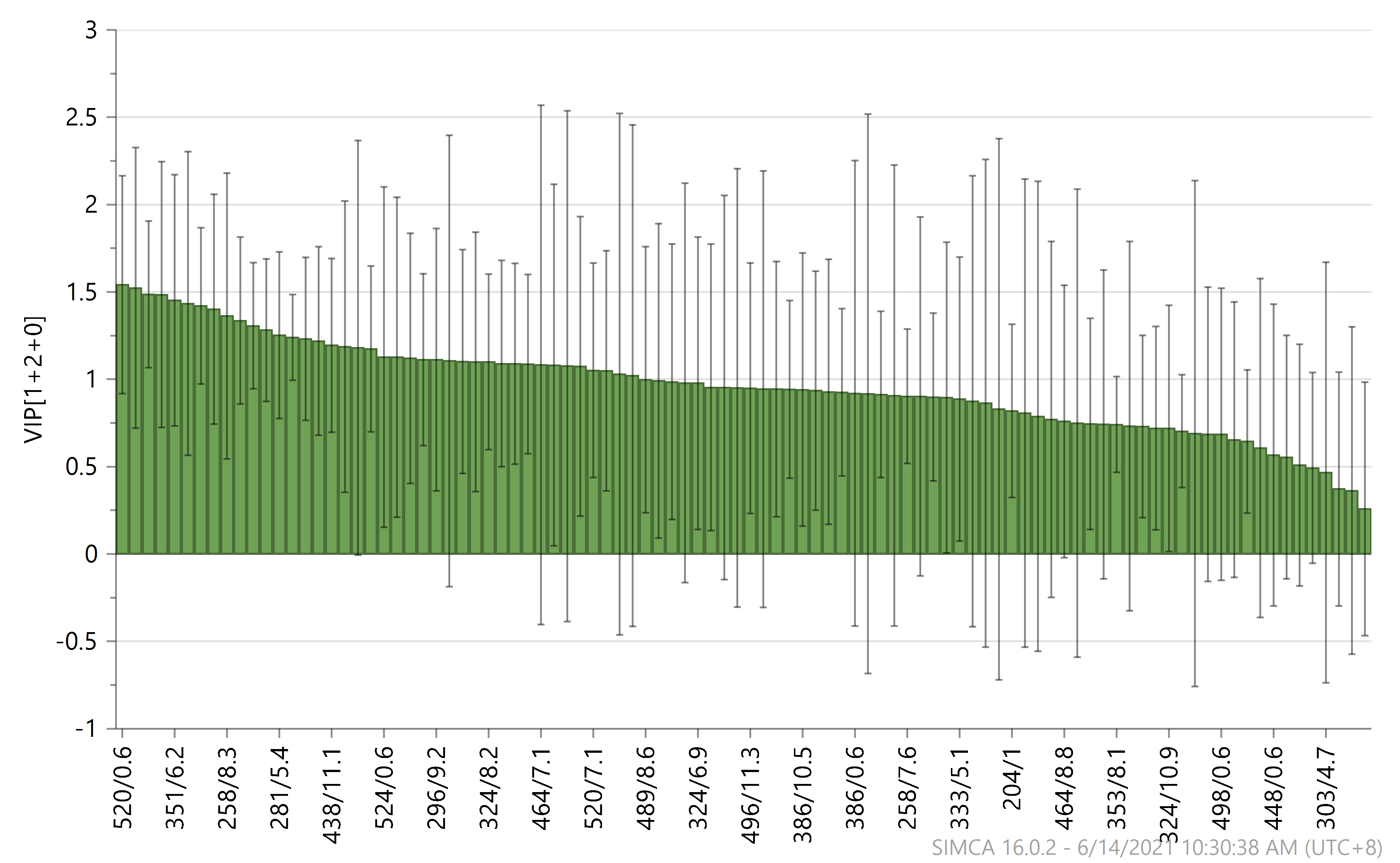


B)


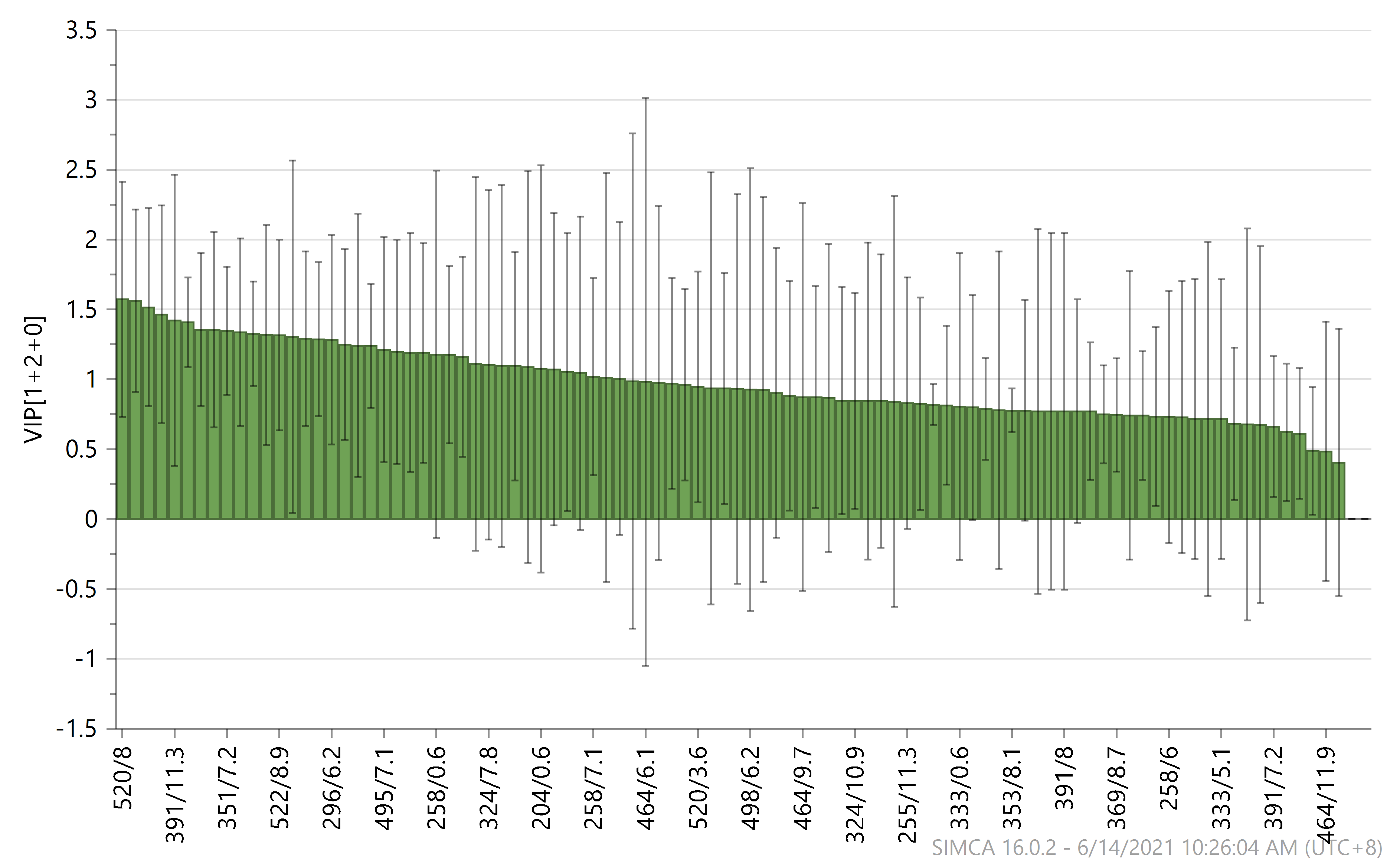


C)


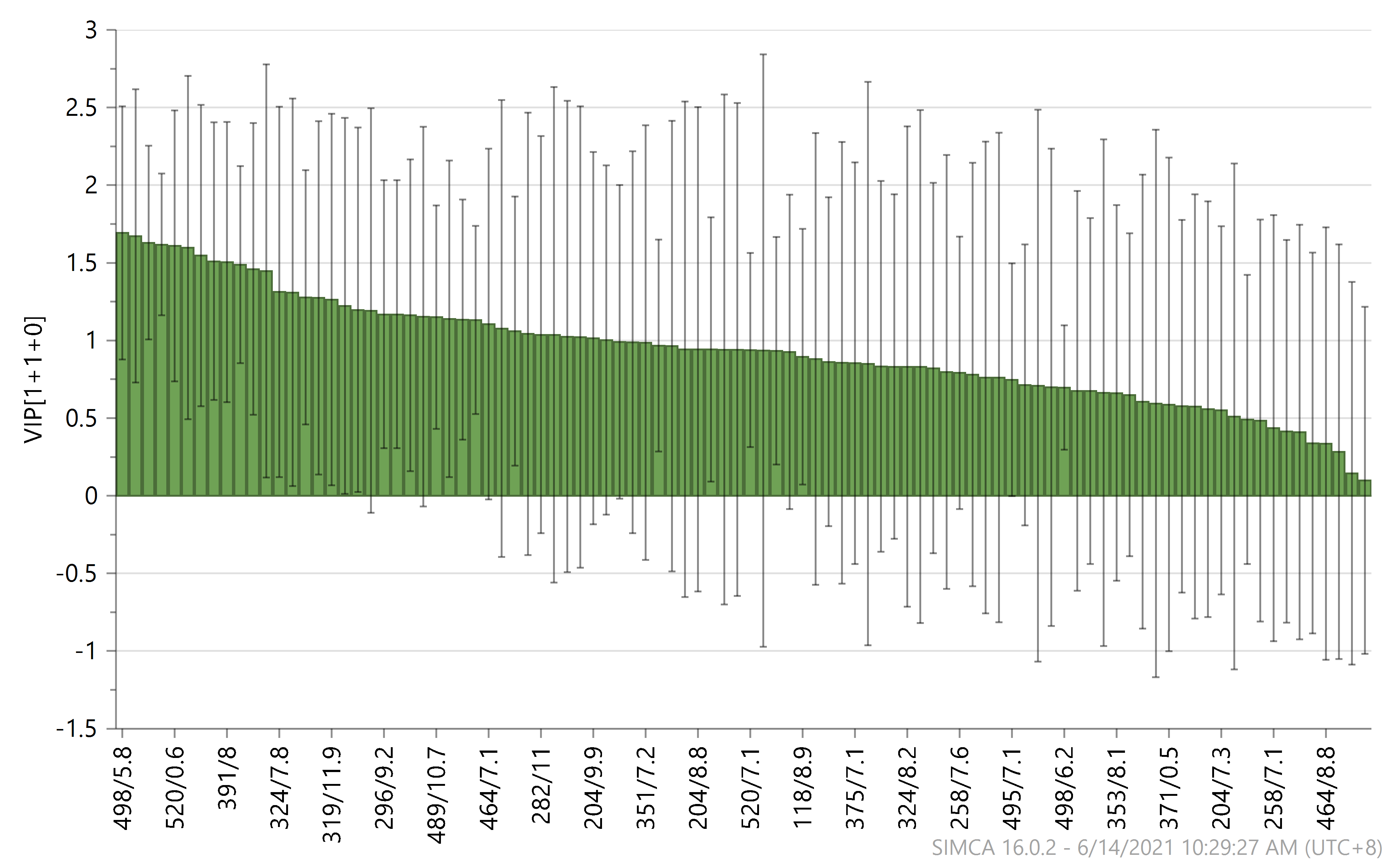


Figure S1: VIP values for OPLS plot for A) control group vs H_2_O_2_ treated cells, B) H_2_O_2_ treated cell vs H_2_O_2_ and avocado seed treated cells, C) control group vs H_2_O_2_ and avocado seed extract treated cells.
